# Adipocytokines and CD34^+^ Progenitor Cells in Alzheimer's Disease

**DOI:** 10.1371/journal.pone.0020286

**Published:** 2011-05-25

**Authors:** Boris Bigalke, Brigitte Schreitmüller, Kateryna Sopova, Angela Paul, Elke Stransky, Meinrad Gawaz, Konstantinos Stellos, Christoph Laske

**Affiliations:** 1 Medizinische Klinik III, Kardiologie und Kreislauferkrankungen, Eberhard-Karls-Universität Tübingen, Tübingen, Germany; 2 Division of Imaging Sciences, School of Medicine, King's College London, The Rayne Institute, London, United Kingdom; 3 Department of Psychiatry and Psychotherapy, University of Tübingen, Tübingen, Germany; Biological Research Center of the Hungarian Academy of Sciences, Hungary

## Abstract

**Background:**

Alzheimer's disease (AD) and atherosclerosis share common vascular risk factors such as arterial hypertension and hypercholesterolemia. Adipocytokines and CD34^+^ progenitor cells are associated with the progression and prognosis of atherosclerotic diseases. Their role in AD is not adequately elucidated.

**Methods and Findings:**

In the present study, we measured in 41 patients with early AD and 37 age- and weight-matched healthy controls blood concentrations of adiponectin and leptin by enzyme linked immunoabsorbent assay and of CD34^+^ progenitor cells using flow cytometry. We found significantly lower plasma levels of leptin in AD patients compared with the controls, whereas plasma levels of adiponectin did not show any significant differences (AD vs. control (mean±SD): leptin:8.9±5.6 ng/mL vs.16.3±15.5 ng/mL;P = 0.038; adiponectin:18.5±18.1 µg/mL vs.16.7±8.9 µg/mL;P = 0.641). In contrast, circulating CD34^+^ cells were significantly upregulated in AD patients (mean absolute cell count±SD:253±51 vs. 203±37; P = 0.02) and showed an inverse correlation with plasma levels of leptin (r = −0.248; P = 0.037).

In logistic regression analysis, decreased leptin concentration (P = 0.021) and increased number of CD34^+^ cells (P = 0.036) were both significantly associated with the presence of AD. According to multifactorial analysis of covariance, leptin serum levels were a significant independent predictor for the number of CD34^+^ cells (P = 0.002).

**Conclusions:**

Our findings suggest that low plasma levels of leptin and increased numbers of CD34^+^ progenitor cells are both associated with AD. In addition, the results of our study provide first evidence that increased leptin plasma levels are associated with a reduced number of CD34^+^ progenitor cells in AD patients. These findings point towards a combined involvement of leptin and CD34^+^ progenitor cells in the pathogenesis of AD. Thus, plasma levels of leptin and circulating CD34^+^ progenitor cells could represent an important molecular link between atherosclerotic diseases and AD. Further studies should clarify the pathophysiological role of both adipocytokines and progenitor cells in AD and possible diagnostic and therapeutic applications.

## Introduction

Increased plasma leptin levels have been found to be associated with a lower risk of incident dementia and Alzheimer's disease (AD) [1]. Cerebrovascular dysfunction is a well-known finding in patients with AD [2], and leptin may be an important therapeutic target [3]. Even though plasma levels of adipocytokines leptin and adiponectin are associated with the progression and prognosis of atherosclerotic diseases showing a significant increase in patients with acute coronary syndrome compared to patients with stable angina pectoris [4], the assessment of adipocytokine plasma levels in AD patients needs further elucidation due to contrasting results of adipocytokine plasma concentrations [5].

AD and atherosclerosis share the same classical cerebro-/cardiovascular risk factors such as hypertension, hyperlipidemia, diabetes mellitus type 2, obesity and smoking [6]. Considering this vascular component in AD allows us to find key aspects including epidemiology, genetics, pathogenesis, diagnosis, and treatment in an analogous view to coronary artery disease (CAD) [7]. Previously, our group has described associations of plasma levels of platelet-derived soluble collagen receptor glycoprotein VI (GPVI) as well as of stromal cell-derived factor 1 (SDF-1) with AD patients [8], [9]. We have recently shown that CD34^+^ progenitor cells are stage-dependently upregulated in AD patients [10], which may reflect vascular repair processes in the brain [11]. Several studies examined assocations of adipocytokines and progenitor cells and focused on their vascular effects in patients with acute myocardial infarction and with metabolic syndrome [12]–[14]. To date, no study has focused on the presence of AD and the potential association between adipocytokines and number of CD34^+^ progenitor cells in AD patients so far. Even though AD neurodegeneration, stroke, and CAD share similar vascular repair mechanisms [15], differential levels and therapeutic effects of progenitor cells in vascular regeneration produced inconsistent results in cardiovascular research [16], [17].

Regarding patients with CAD, differential plasma concentrations of adiponectin have been controversially discussed for the predictive value [4], [18]–[20]. Moreover, adiponectin seems to act in a protective way in comorbidities such as diabetes mellitus type 2, insulin resistance, metabolic syndrome, and inflammation [21]–[23], whereas correlations of leptin to classical risk markers such as troponin-I and C-reactive protein may reflect the degree of inflammation in the process of plaque instability [24].

The aim of this study was to differentially evaluate AD presence and find associations between the plasma levels of both adipocytokines (leptin and adiponectin) and their influence on the number of CD34^+^ progenitor cells reflecting the initiation of vascular healing process in the brain in patients with AD.

## Methods

### Subjects

We consecutively evaluated 41 patients with early AD from our Memory Clinic at the University Hospital of Psychiatry and Psychotherapy Tuebingen and compared them to 37 healthy elderly controls. Patients' demographic and clinical details are presented in *Table 1*.

**Table 1:** Patients' Characteristics and Premedication on Hospital Admission.

Characteristics	All(n = 78)	AD(n = 41)	Control(n = 37)	*P* Value(AD vs. Control)
**Age – years**	71±10.2	74.3±9.1	67.3±10.2	0.598
**Sex – no. (%)**				0.415
**Female**	40 (51.3)	22 (53.7)	18 (48.6)	
**Male**	38 (48.7)	19 (46.3)	19 (51.4)	
**Cerebro-/cardiovascular risk factors – no. (%)**				
**Arterial hypertension**	33 (42.3)	18 (43.9)	15 (40.5)	0.472
**Hyperlipidaemia**	28 (35.9)	15 (36.6)	13 (35.1)	0.442
**Diabetes mellitus**	6 (7.7)	3 (7.3)	3 (8.1)	0.612
**Family history of CAD**	9 (11.5)	6 (14.6)	3 (8.1)	0.295
**Smoking**	10 (12.8)	7 (17.1)	3 (8.1)	0.201
**Obesity (BMI≥30)**	16 (20.5)	6 (14.6)	10 (35.4)	0.271
**Comorbidities**				
**Coronary artery disease (CAD)**	12 (15.4)	7 (17.1)	5 (13.5)	0.454
**History of myocardial Infarction/stroke**	7 (8.9)	4 (23.1)	3 (8.1)	0.558
**Mini-mental state examination score (MMSE)**	24.5±5.8	19.9±4.6	29.4±0.6	0.001
**Premedication – no. (%)**				
**ACE Inhibitors**	31 (39.7)	17 (41.5)	14 (37.8)	0.463
**Statins**	17 (21.8)	11 (26.8)	6 (16.2)	0.196
**NSAID**	20 (25.6)	13 (31.7)	7 (18.9)	0.151

*mean ± standard deviation. AD denotes Alzheimer's disease, CAD coronary artery disease, BMI body mass index, ACE angiotensin converting enzyme, NSAID non-steroidal anti-inflammatory drugs.

Patients with AD fulfilled the criteria of ICD-10, DSM-IV and the National Institute of Neurologic and Communicative Disorders and Stroke and the Alzheimer's Disease and Related Disorders Association (NINCDS-ADRDA) for probable AD [25]. The clinical severity of cognitive impairment was assessed by the mini-mental state examination (MMSE) [26].

AD patients or control subjects with current or a history of depression or psychosis, with major physical illness, alcohol or substance abuse or use of psychoactive medications were excluded from the study.

The study was performed according to the ethical principles of the Declaration of Helsinki (sixth revision, 2008) and was approved by the local ethics committee of the University Hospital Tuebingen. We obtained written informed consent from all subjects participating at the study (in case of AD patients: by themselves or by legally authorized representatives).

### Blood sampling

Blood samples were obtained in the morning (9.00–10.00 A.M.; in the fasting state). Venous blood was filled into 5 mL ethylenediaminetetraacetic acid (EDTA) plasma probes for the determination of baseline concentrations of adiponectin, and leptin using an enzyme-linked immunosorbent assay (ELISA) kit as well as into 3.8% citrate plasma tubes for the peripheral blood monocnuclear cell isolation.

### Flow cytometry

According to previous protocols, mononuclear cells were isolated using a Ficoll density gradient (Biocoll, Biochrom, Berlin, Germany) [10], [27]. Mononuclear cells were resuspended in 100 µl of phosphate buffered saline. For flow cytometric analyis, we used Fluorescein (FITC)-conjugated anti-CD34 antibodies (Becton Dickinson, San Jose, USA; clone 8G12) and IgG1-FITC (BD Biosciences Pharmingen, USA; clone MOPC-21) served as negative isotype control. Each measurement was performed in duplicate. After 250,000 events have been reached in a lymphocyte gate, we used the absolute cell counts as units.

### *ELISA*

Plasma levels of adiponectin and leptin were determined in a total group of 78 consecutive AD patients and healthy controls using a commercially available ELISA kit according to the manufacturer's guidelines (R&D Systems, Minneapolis, MN, USA). EDTA plasma probes were centrifuged for 15 minutes at 10,000 g within 30 minutes of collection. Probes were aliquotted and stored at −20°C before analysis. Lower detection limits of these assays were 15.6 pg/mL for leptin and 0.079 ng/mL for adiponectin. These assays recognize recombinant and natural leptin and recombinant and natural (low, middle and high molecular weight) human total adiponectin.

### Data analysis

Data are presented as mean ± standard deviation (SD). All tests were two-tailed and statistical significance was considered for P values less than 0.05. For corrections of multiple testing, a Bonferroni–Holm correction was applied. A multiple logistic regression analysis implementing an automatic stepwise selection algorithm for risk factor inclusion was performed to assess independent association of parameters with the presence of AD. Adjustment by possible confounders was performed by the multifactorial analysis of covariance for the decadic logarithm of plasma levels of leptin and adiponectin and the number of CD34^+^ progenitor cells, respectively. The patients have been matched according to age, sex, body weight, classical cerebro-/cardiovascular risk factors, comorbidities, and medical treatment regarding angiotensin converting enzyme (ACE) inhibitors, statins, and non-steroidal anti-inflammatory drugs. Continuous variables were tested for normal distribution with the Kolmogorov-Smirnov test. The two-tailed t-test was used to assess differences between two groups in case of normal distribution. The Mann-Whitney U-test was used to assess differences between two groups in case of non-normal distribution. Comparison of categorical variables was generated by the Pearson chi-square test. Correlations were assessed with the Spearman correlation coefficient test of the data. All statistical analyses were performed using computer software program SPSS version 15.0.1 for windows (SPSS Inc., Chicago, IL, USA).

## Results

We consecutively evaluated 41 patients with early AD (22 women, 19 men; mean age ± SD: 74.3±9.1 years) and compared them to 37 healthy elderly controls (18 women, 19 men; mean age ± SD: 67.3±10.2 years). AD patients showed a mean MMSE score ± SD of 19.8±4.5. The control group had a normal cognitive status according to clinical examination and MMSE score (mean MMSE score ± SD: 29.4±0.6). Patients' demographic and clinical details are presented in *Table 1*.

**Figure 1:**
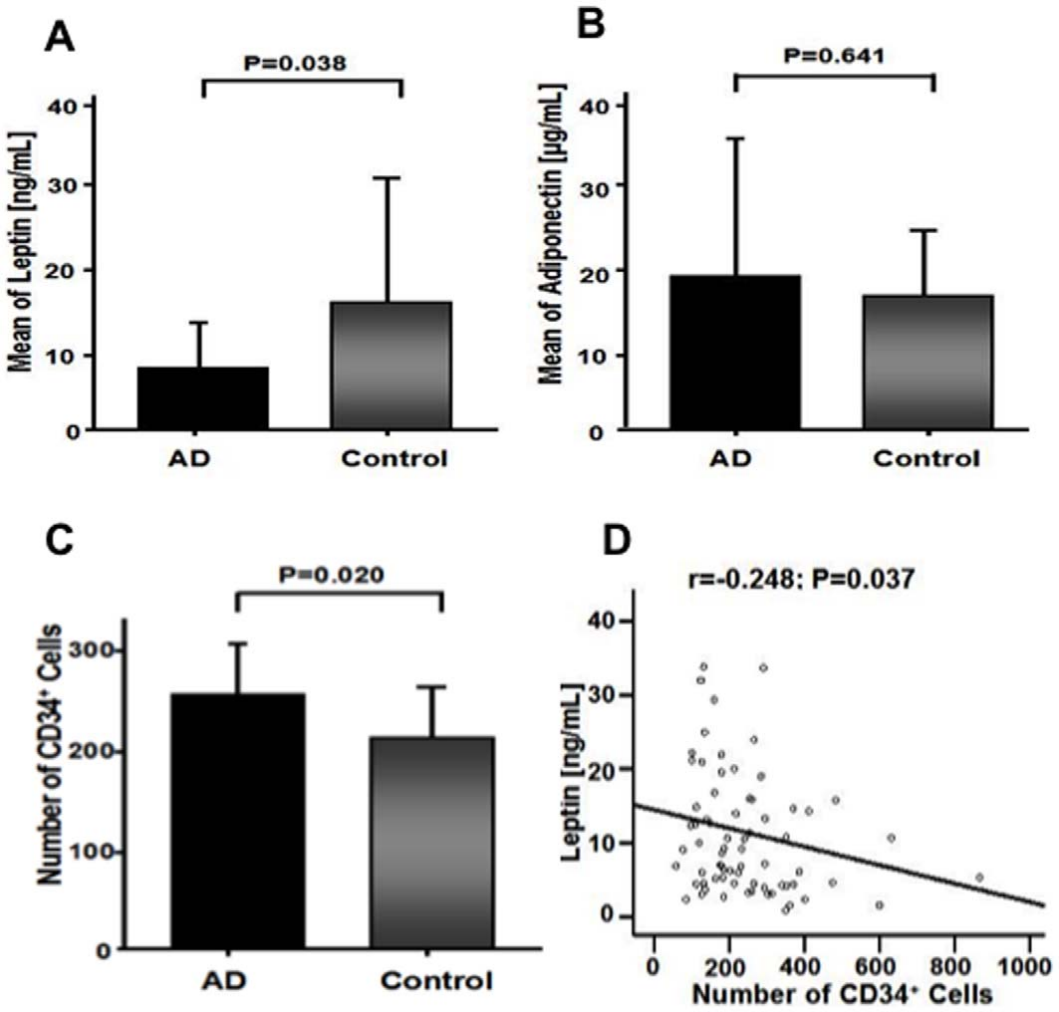
Plasma levels of adipocytokines leptin and adiponectin, and number of CD34^+^ progenitor cells in Alzheimer's disease (AD). (A) AD patients showed significantly lower plasma leptin levels compared with healthy controls (P = 0.038). (B) Plasma levels of adiponectin did not show any significant differences between AD patients and healthy controls (P = 0.641). (C) Number of CD34^+^ progenitor cells [mean absolute cell count] was significantly upregulated in patients with AD compared to control (P = 0.02). (D) Plasma levels of leptin inversely correlated with the number of CD34^+^ progenitor cells (r = −0.248; P = 0.037).

We found significantly lower plasma levels of leptin in AD patients compared with healthy controls *(* *Fig. 1A* *)*, whereas plasma levels of adiponectin did not show any significant differences (AD vs. control (mean±SD): leptin: 8.9±5.6 ng/mL vs. 16.3±15.5 ng/mL; P = 0.038; adiponectin: 18.5±18.1 µg/mL vs. 16.7±8.9 µg/mL; P = 0.641) *(* *Fig. 1B* *)*. However, plasma levels of both adipocytokines significantly correlated with each other (r = 0.402; P = 0.001) in the combined sample pool of AD patients and controls.

The number of circulating CD34^+^ progenitor cells were significantly upregulated in AD patients (mean absolute cell count±SD: 253±51 vs. 203±37; P = 0.02) *(* *Fig. 1C* *)*. Moreover, we found a significant, inverse correlation with the plasma levels of leptin (r = −0.248; P = 0.037) *(* *Fig. 1D* *)*.

To test whether plasma levels of adipocytokines (leptin, adiponectin) and CD34^+^ progenitor cells are independently associated with the presence of AD, we performed a multivariate logistic regression analysis including parameters such as age, gender, classical cerebro-/cardiovascular risk factors, comorbidities, and medication. Among the variables tested, plasma levels of leptin were negatively (P = 0.021) and number of CD34^+^ progenitor cells positively (P = 0.036) associated with the presence of AD (*Table 2* *)*.

**Table 2:** Multivariate Logistic Regression Modeling.

Parameters Tested	Regression Coefficient β	*P* Value
Leptin	−0.207	0.021
Adiponectin	−0.039	0.108
CD34^+^ Cells	0.115	0.036
Age	−0.335	0.142
Gender (Male)	−0.196	0.347
Arterial hypertension	−0.048	0.543
Hyperlipidemia	0.133	0.072
Diabetes mellitus	0.231	0.081
Family history of CAD	0.062	0.369
Smoking	0.109	0.171
Body mass index	0.322	0.207
Coronary artery disease (CAD)	0.008	0.558
History of myocardial infarction/stroke	0.121	0.484
ACE Inhibitors	−0.013	0.255
Statins	0.285	0.093
NSAID	0.024	0.243

Comparisons of the decadic logarithm of plasma levels of leptin, adiponectin and CD34^+^ cells between AD and controls were adjusted by possible confounders such as age, sex, body weight, classical cerebro-/cardiovascular risk factors, comorbidities, and medical treatment. Thus, plasma levels of leptin and CD34^+^ cells have been independently associated with AD compared to controls (leptin: P = 0.002; CD34^+^ cells: P = 0.022), whereas adiponectin plasma levels neither have been associated with AD (P = 0.470) nor have been influenced by any other potential confounders (all,P>0.05) *(* *Table 3* *)*.

**Table 3:** Multifactorial Analysis of Covariance.

Category	Factor	*P* ValueLeptin	*P* ValueAdiponectin	*P* ValueCD34^+^ Cells
Age				
	Years	0.289	0.654	0.273
Sex				
	Male vs. Female	0.445	0.189	0.108
Cerebro-/cardiovascular risk factors				
	Arterial hypertension	0.612	0.223	0.435
	Hyperlipidemia	0.905	0.418	0.503
	Diabetes mellitus	0.964	0.562	0.700
	Family history of CAD	0.556	0.701	0.932
	Smoking	0.698	0.577	0.629
	Body mass index	0.279	0.176	0.194
Comorbidities				
	Coronary artery disease (CAD)	0.834	0.314	0.341
	History of myocardial infarction/stroke	0.656	0.165	0.560
Medication				
	ACE Inhibitors	0.467	0.834	0.319
	Statins	0.948	0.923	0.666
	NSAID	0.655	0.571	0.205
Groups				
	AD vs. Control	0.002	0.470	0.022

**Figure 2:**
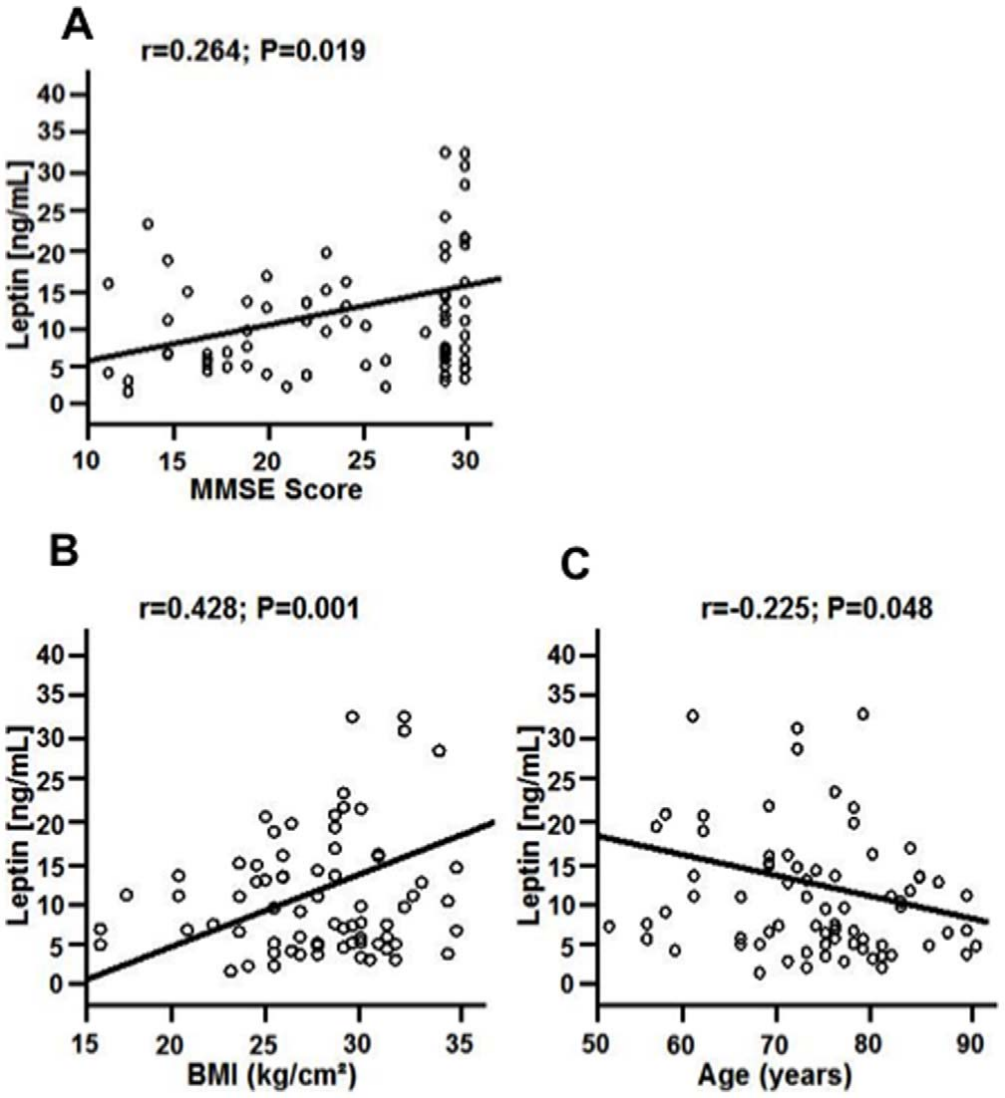
Correlation of baseline concentrations of leptin according to mini-mental state examination (MMSE) score. (A) Lower plasma levels of leptin significantly correlated with a lower MMSE score (r = 0.264; P = 0.019). (B) High leptin plasma levels positively correlated with an increased body mass index (BMI) (r = 0.428; P = 0.001) and (C) inversely correlated with advancing age (r = −0.225; P = 0.048).

Plasma levels of leptin significantly correlated with the severity of AD according to MMSE score (r = 0.264; P = 0.019) *(* *Fig. 2A* *)*, whereas increased plasma levels of adiponectin showed an inverse trend to a lower MMSE score, although this did not reach a statistically significant level (r = −0.137; P = 0.062). Moreover, high leptin plasma levels positively correlated with an increased body mass index (BMI) (r = 0.428; P = 0.001) *(* *Fig. 2B* *)*, and inversely correlated with advancing age (r = −0.225; P = 0.048) *(* *Fig. 2C* *)*. However, high adiponectin levels did not correlate with BMI (r = 0.119; P = 0.314) or with age, respectively (r = 0.099; P = 0.396).

## Discussion

The major findings of the present observational study are: 1) AD patients had significantly decreased plasma levels of leptin compared with healthy controls, whereas circulating CD34^+^ cells were significantly upregulated in AD patients; 2) in logistic regression analysis, decreased leptin concentration and increased number of CD34^+^ cells were both significantly associated with the presence of AD; 3) high plasma levels of leptin inversely correlated with a lower MMSE score and an advancing age and positively correlated with an increase in BMI.

Adipocytokines leptin and adiponectin are associated with the incidence, progression and outcome of atherosclerotic diseases, although previous studies have provided contrasting results about differential plasma concentrations of the adipocytokines in CAD, ischemic stroke and AD [4], [5], [18]–[20], [28], [29]. Recently, circulating leptin has been described to be associated with reduced incidence of dementia and AD and with a higher total cerebral brain volume determined by magnetic resonance imaging in asymptomatic older adults [1]. These results suggesting a protective role of leptin were supported by our findings with decreased baseline plasma levels of leptin in AD patients compared to healthy controls. Furthermore, a decrease of circulating leptin may also have a detrimental effect on the severity of cognitive dysfunction as determined by MMSE score.

To the best of our knowledge, the potential association between adipocytokines and the number of progenitor cells has not been reported so far in AD patients. Several *in vivo* experiments have shown that leptin may significantly increase the recruitment of hematopoetic as well as endothelial progenitor cells and promote vascular regeneration after vascular injury [30]–[32]. As an angiogenic factor, leptin induces neovascularization as well as vascular permeability [33], [34]. Moreover, leptin may regulate hippocampal progenitor cells enhancing neurogenesis in adult mice [35]. In AD patients, we have recently reported increased number of circulating CD34^+^/CD133^+^ progenitor cells in patients with moderate to severe dementia [10]. In the present study, we could demonstrate an inverse correlation between decreased plasma leptin levels and increased circulating CD34^+^ progenitor cells. This finding is in line with the results of a recent study in patients with obesity and could be due to a negative feedback mechanism of pro-angiogenic factors [13].

Although plasma levels of adiponectin have been frequently examined in previous studies for CAD and ischemic stroke [4], [15]–[17], [26], none has focused on patients with AD so far. Thus, we found that adiponectin has neither been associated with AD nor has been influenced by any other possible confounders. However, plasma levels of adiponectin are inversely correlated to MMSE scores, although this did not reach a statistically significant level. Of interest, plasma levels of both adipocytokines significantly correlated with each other, which has been described before in patients with CAD [4]. It is well-known that pathological changes in the brain and cognitive dysfunction are associated with an age-related decline [36]. In addition, age is known as the major risk factor of AD [36]. Interestingly, we found a significant inverse correlation between leptin plasma levels and age in the whole study population of AD patients and healthy controls. This result indicates that leptin plasma levels decrease with advancing age, possibly in a continuum that culminates with AD. Furthermore, weight loss often precedes dementia in AD patients [37] and BMI, hyperlipidemia, and diabetes mellitus have a significant impact on the expression of leptin and adiponectin [37], [38]. However, associations of leptin and BMI or MMSE score produced inconsistent results, which, in some cases, may be explained with leptin resistance in obese humans [39]–[41]. Our collective showed that high leptin plasma levels positively correlated with an increased BMI.

Previous studies have revealed how leptin may be directly associated with AD pathology in the brain [1], [6], [42]. Two major hallmarks of the molecular pathogenesis of AD are accumulation of amyloid-beta (Aβ) peptides to amyloid plaques and deposition of hyperphosphorylated tau proteins to neurofibrillary tangles [6]. Recent experimental studies with animal models of AD have shown that cholesterol-enriched diets and cholesterol metabolites increase Aβ and phosphorylated tau levels in the brain by reducing leptin levels [42]. Thus, treatment with leptin reversed the 27-OHC-induced increase in Aβ and phosphorylated tau by decreasing the levels of β-secretase (BACE-1) and glycogen synthetase kinase-3β (GSK-3β) respectively [42]. The protective effect of leptin administration against AD pathology in the brain has also been demonstrated in other experimental studies [43]. These experimental findings indicate that leptin administration could be a promising new treatment strategy against AD.

Furthermore, adipocytokines may contribute as a diagnostic tool to a multimarker strategy in AD simultaneously evaluating biomarkers such as endothelin-1, atrial natriureticpeptide, and adrenomedullin with immune modulating, metabolic, and vascular characteristics [44]. The development and assessment of a multimarker panel of platelet activity, vascular repair and tissue regeneration could be worthwhile, as we have previously found associations of plasma levels of platelet-derived soluble GPVI, SDF-1, and CD34^+^/CD133^+^ progenitor cells with AD patients [8]–[10].

In conclusion, our findings suggest that low plasma levels of leptin and increased numbers of CD34^+^ progenitor cells are both associated with AD. In addition, the results of our study provide first evidence that increased leptin plasma levels are associated with a reduced number of CD34^+^ progenitor cells in AD patients. These findings point towards a combined involvement of leptin und CD34^+^ progenitor cells in the pathogenesis of AD. Thus, plasma levels of leptin and circulating CD34^+^ progenitor cells could represent an important molecular link between atherosclerotic diseases and AD. Further studies should clarify the pathophysiological role and interaction of both adipocytokines and progenitor cells in AD and possible diagnostic and therapeutic applications.
